# Extracorporeal Shock Wave Treatment (ESWT) Improves *In Vitro* Functional Activities of Ruptured Human Tendon-Derived Tenocytes

**DOI:** 10.1371/journal.pone.0049759

**Published:** 2012-11-26

**Authors:** Laura Leone, Mario Vetrano, Danilo Ranieri, Salvatore Raffa, Maria Chiara Vulpiani, Andrea Ferretti, Maria Rosaria Torrisi, Vincenzo Visco

**Affiliations:** 1 Department of Clinical and Molecular Medicine, Istituto Pasteur-Fondazione Cenci Bolognetti, Sapienza University of Rome, Rome, Italy; 2 Department of Ortophaedics and Traumatology, Sapienza University of Rome, Rome, Italy; 3 Sant’Andrea Hospital, Rome, Italy; The University of Tennessee Health Science Center, United States of America

## Abstract

*In vitro* models of human tenocytes derived from healthy as well as from ruptured tendons were established, characterized and used at very early passage (P1) to evaluate the effects of Extracorporeal Shock Wave Treatment (ESWT). The molecular analysis of traditional tenocytic markers, including Scleraxis (Scx), Tenomodulin (Tnm), Tenascin-C (Tn-C) and Type I and III Collagens (Col I and Col III), permitted us to detect in our samples the simultaneous expression of all these genes and allowed us to compare their levels of expression in relationship to the source of the cells and treatments. In untreated conditions, higher molecular levels of Scx and Col I in tenocytes from pathological compared to healthy samples have been detected, suggesting – in the cells from injured tendon – the natural trigger of an early differentiation and repairing program, which depends by Scx and requires an increase in collagen expression. When ESWT (at the dose of 0.14 mJ/mm^2^) was applied to cultured tenocytes explanted from injured source, Scx and Col I were significantly diminished compared to healthy counterpart, indicating that such natural trigger maybe delayed by the treatment, in order to promote cellular repair. Herein, we show for the first time that ESWT enhances *in vitro* functional activities of ruptured tendon-derived tenocytes, such as proliferation and migration, which could probably contributes to tendon healing *in vivo*.

## Introduction

Despite some advancements in the management of acute and chronic tendinopathies, several aspects related to the pathophysiology of human tendons remain largely unclear [1]–[3]. Tendon cells, residing between fibril components mainly represented by collagens and proteoglycans, synthetize extracellular matrix (ECM) [4]–[6].

Several *in vitro* models of human cultured tenocytes have been useful for better understanding the cellular and molecular mechanisms involved in tendon degeneration, in order to develop novel therapies for the management of tendinopathies [7]–[12]. Maffulli et al. [7] demonstrated that human tenocytes from ruptured tendons -compared to healthy counterparts- contained reduced amounts of type I (and increased of type III) collagen, which is known to be the main content of ECM in normal tissue. Besides type I and III collagens, tenocytes are known to express a few traditional molecular markers, including Scleraxis (Scx), Tenomodulin (Tnm) and Tenascin-C (Tn-C), whose combination is commonly used to characterize tendon cells [12]–[19]. Scx is a transcriptional regulator detected in progenitors of tendon cells as well as in differentiated tenocytes [15], [20], [21], which is crucial for subsequent expression of Tnm [15], a type II transmembrane protein selectively expressed in more mature tenocytes. Tn-C represents an additional marker which is reliable of the tenocytic lineage, although it has been also found in cartilage and nerve [15], [17]. Except for type I and III collagen production, to date and to our knowledge, no significant differences in molecular expression of such tendon cell markers from normal versus pathological tissues have been yet documented.

Several reports investigated the rationale of applying *in vivo* Extracorporeal Shock Waves Treatment (ESWT) on tendon tissue using *in vitro* models [22], [23]. We have recently reported that the ESWT of human cultured tenocytes is able to induce *in vitro* cell proliferation and synthesis of type I collagen, both appear being more significant from 8 to 12 days following exposure [24]. Because only tenocytes derived from healthy tendons have been used in that study, here we aimed to clarify whether tenocytes from pathological tendons may display different gene expression and functional behaviour following ESWT. To this purpose, here we first compared the cultures of human tenocytes explanted by ruptured Achilles versus healthy semitendinosus tendons, both used at the same very early passage (P1). Then, the *in vitro* ESWT-induced effects were evaluated on the different cultures of human tenocytes, analysing the repairing activity of the tendons either by the expression of critical genes or the functional outcome on cell proliferation and motility.

## Materials and Methods

### Tissue Samples, Primary Cultures of Human Tenocytes and Treatments

Biopsies were obtained from eight patients (5 men and 3 women aged from 15 to 53 yrs): four from semitendinosus tendon, in patients that underwent arthroscopic Anterior Cruciate Ligament (ACL) reconstruction and four from Achilles tendon, in patients receiving surgery for rupture of the Achilles tendon and all previously affected by a pre-existing history of chronic degenerative tendinopathy.

The Institutional Review Board of ‘‘Sapienza’’ University and Sant’Andrea Hospital approved the study protocol, and all patients gave their written informed consent to the experimental study.

Tissue biopsies were cut into small pieces (2.5–3.0 mm^3^) and digested with 2 mg/ml collagenase type I (GIBCO). The samples were centrifuged at 1000 rpm for 10 minutes: the supernatants were discarded and the cell pellets were cultured in D-MEM, supplemented with 10% fetal bovine serum and antibiotics.

Primary cultures were expanded for one passage (P1) and were used for the following experiments.

In order to assess the shock wave effects, at passage P1 primary cultured tenocytes were divided into two groups: ESWT treatment group (connected to the shock wave generator, as previously described [25] and ESWT non-treatment group as control.

The shock wave treatment was applied using an electromagnetic shock wave generator MODULITH® SLK (STORZ MEDICAL AG; Tägerwilen, Switzerland), at a dose of shock waves 0.14 mJ/mm^2^ energy level and 1000 impulses as previously described [24], whereas the control group was maintained in the same culture conditions, without previous shock wave exposure. Treated and untreated cells were incubated at 37°C and used for the following experiments from day 1 to day 4.

### Flow Cytometry for Cell Cycle

For each culture the cells were plated sparsely at P1 so that they did not touch each other and did not reach contact inhibition. Cells were trypsinized, pelleted and resuspended in 70% ethanol in PBS, and stored at 4°C overnight. Cells were washed with PBS, resuspended in propidium iodide (PI) staining solution (50 µg/ml) and RNAse A (100 Kunitz/ml) (Miltenyi Biotec GmbH, Bergisch Gladbach, Germany) and incubated in the dark for 40 minutes at room temperature. At least 20,000 cells were collected and analyzed with MACSQuant® Analyzer flow cytometer (Miltenyi Biotec GmbH). Cell cycle distribution was calculated with MACSQuantify® software (Miltenyi Biotec GmbH).

### Primers

Oligonucleotide primers for target genes and for the housekeeping gene (18 s) were chosen with the assistance of the Oligo 5.0 computer program (National Biosciences, Plymouth, MN) and purchased from Invitrogen. The primers used are listed in Table 1. For each primer pair, we performed no-template control and no-reverse-transcriptase control (RT negative) assays, which produced negligible signals.

**Table 1:** Primers used for target and housekeeping genes.

Gene	Primer sequence (5′-3′)Forward-Reverse
Col I	5′-ACATGTTCAGCTTTGTGGACCTCCG-3′ 5′-ACGCAGGTGATTGGTGGGATGTCT-3′
Col III	5′-AGGGTGTCAAGGGTGAAAGTGGGA-3′ 5′-ACCAGCCAGACCAGGAAGACCC-3′
Scx	5′-CGAGCGAGACCGCACCAACA-3′ 5′-CGTTGCCCAGGTGCGAGATGTAG-3′
Tnm	5′-CCGCCGCGTCTGTGAACCTT-3′ 5′-GCGGGCCACCCACCAGTTAC-3′
Tn-C	5′-GGAGGGGACCACGCTGAGGT-3′ 5′-TCCCGGCCTCAGACCTGTGAG-3′
18 s	5′-CGAGCCGCCTGGATACC-3′ 5′-CATGGCCTCAGTTCCGAAAA-3′

### RNA Extraction and cDNA Synthesis

RNA was extracted using the TRIzol method (Invitrogen, Carlsbad, CA) according to manufacturer’s instructions and eluted with 0.1% diethylpyrocarbonate (DEPC)-treated water. Total RNA concentration was quantitated by spectrophotometry and the quality was assessed by measuring the optical density ratio at 260/280 nm. RNA samples were stored at −80°C. After denaturation in DEPC-treated water at 70°C for 10 min, 1 mg of total RNA was used to reverse transcription using iScript™ cDNA synthesis kit (Bio-Rad) according to manufacturer’s instructions.

### PCR Amplification and Real-time Quantitation

Real-time PCR was performed using the iCycler Real-Time Detection System (iQ5 Bio-Rad) with optimized PCR conditions. The reaction was carried out in 96-well plate using iQ SYBR Green Supermix (Bio-Rad) adding forward and reverse primers for each gene and 1 ml of diluted template cDNA to a final reaction volume of 15 ml. All assays included a negative control and were replicated three times. The thermal cycling programme was performed as follows: an initial denaturation step at 95°C for 3 min, followed by 45 cycles at 95°C for 10 sec. and 60°C for 30 sec. Real-time quantitation was performed with the help of the iCycler IQ optical system software version 3.0 a (Bio-Rad), according to the manufacturer’s manual. The relative expression of the housekeeping gene was used for standardizing the reaction. The comparative threshold cycle (*C*t) method was applied to calculate the fold changes of expression compared to control cells. Results are reported as mean±standard deviation (SD) from three different experiments in triplicate.

### Proliferation Assay

For BrdU incorporation assay to evaluate DNA synthesis and cell proliferation, cells exposed to ESWT as above were plated on coverslips and incubated for 96 h at 37°C. Unexposed cells were used as control. During the last 4 h cells were incubated with 100 mM BrdU (Sigma) added to the medium. Cells were then fixed in 4% formaldehyde in phosphate buffered saline (PBS) for 30 min at 25°C, followed by treatment with 0.1 M glycin for 20 min at 25°C and with 0.5% HCl, 0.1% triton X-100 for additional 45 min at 25°C to allow permeabilization. After extensive washing in PBS, cells were buffered with 0.1 M Na_2_B_4_O_7_ and incubated with anti-BrdU monoclonal antibody (1∶50 in PBS) (Sigma) for 30 min at 25°C, followed by goat anti-mouse immunoglobulin G–FITC (1∶20 in PBS) (Cappel Research Products, Durham, NC, USA). Nuclei were stained with 4,6-diamidino-2-phenylindole (DAPI 1∶10 000 in PBS; Sigma). Coverslips were finally mounted with mowiol for observation. Fluorescence signals were analyzed by conventional fluorescence or by scanning cells in a series of 0.5 µm sequential sections with an ApoTome System (Zeiss, Oberkochen, Germany) connected with an Axiovert 200 inverted microscope (Zeiss); image analysis was then performed by the Axiovision software (Zeiss).

Percentage of BrdU positive cells were analyzed counting for each treatment a total of 500 cells, observed in 10 microscopic fields randomly taken from three different experiments. Results have been expressed as mean values±SE.

### Scratch Assay

Cells exposed to ESWT were seeded on 24 well plates and incubated for 48 h at 37°C. Then a standardized cell-free area was introduced by scraping the monolayer with a sterile tip, as previously described [26]. Unexposed cells were used as control. After intensive wash, the remaining cells were incubated for 24 h, fixed with 4% paraformaldehyde for 30 min at 25°C and photographs were taken using an Axiovert 200 inverted microscope (Zeiss). Some plates were fixed and photographed immediately after scratching representing a T0 control. Migration was quantitated by a measure of the recovered scratch area, performed using the Axiovision software (Zeiss). The data presented are a mean of triplicate experiments±SD.

### Statistical Methods

To compare variables that do not assume Gaussian distribution, Mann-Whitney non-parametric test was used. The data are represented with the Tukey box-and-whisker plot, where the central box represents the values from the lower to upper quartile (25^th^ to 75^th^ percentile), the middle line represents the median, and the horizontal lines represent the minimum and the maximum value of observation range. To compare variables that assume Normal distribution, Student’s T test was used. The values are expressed as mean±SD from three independent experiments. P values<0.05 were assumed as statistically significant.

## Results

### Characterization of Primary Cultured Human Tenocytes Derived from Healthy and Ruptured Tendons

**Figure 1:**
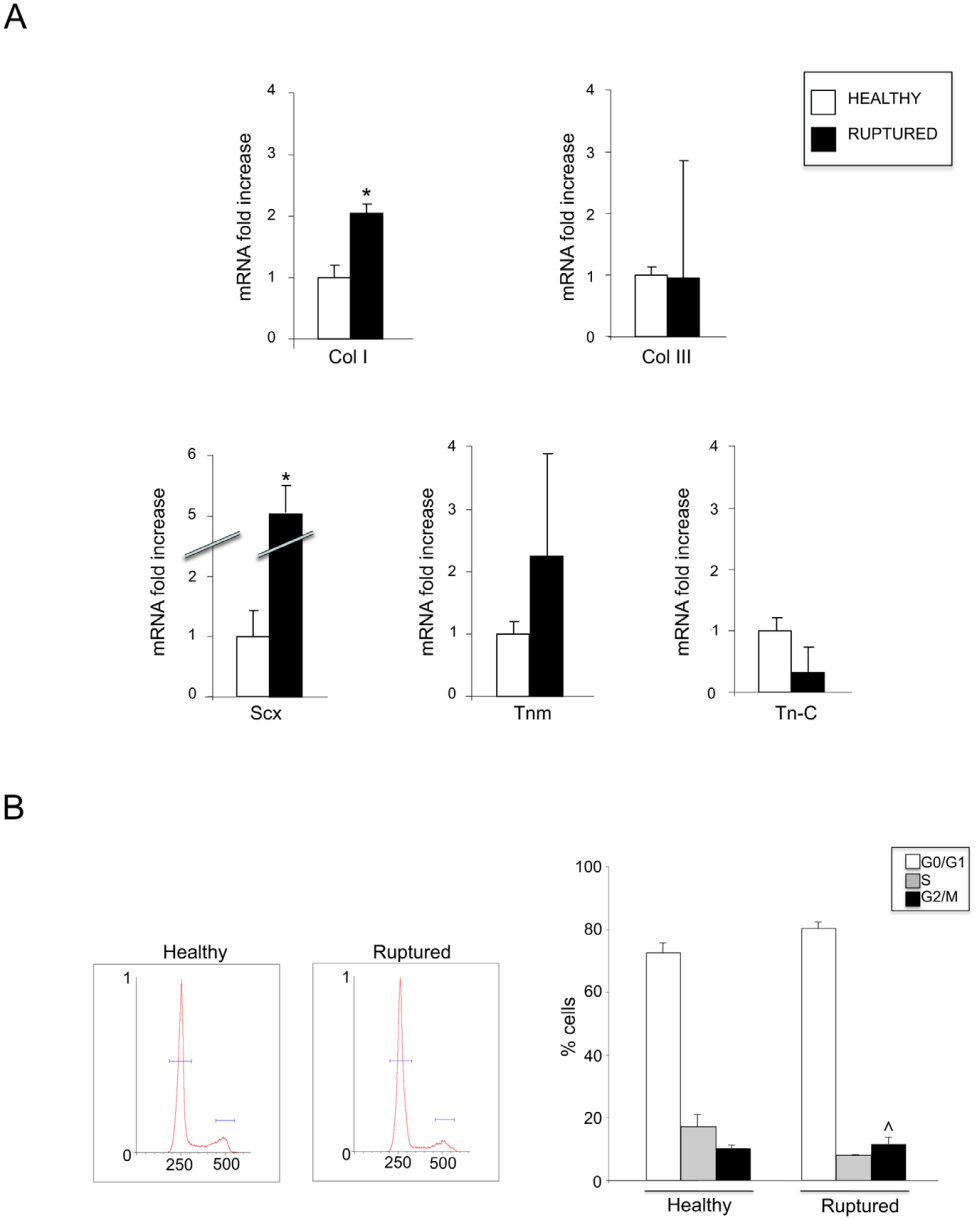
Characterization of the primary cultures of tenocytes derived from healthy and ruptured tendons. A. Molecular analysis of the expression of typical tenocyte markers: Collagen I (Col I), Collagen III (Col III), Scleraxis (Scx), Tenomodulin (Tnm) and Tenascin-C (Tn-C). mRNA transcript levels were quantitated by real-time RT PCR. All markers are expressed in both healthy and ruptured tendon-derived cultures. Significantly higher levels of Col I and Scx mRNA are found in cultures obtained from ruptured respect to healthy tendons while no significant differences are detectable for the other markers (Col III, Tnm, Tn-C). Results reported in graph represent the mean values ±SE obtained from three independent experiments. Student T test was performed and significance level has been defined as described in materials and methods: *p>0.05 vs healthy. B. Tenocytes cultures were stained with PI and analyzed for cell cycle distribution by flow cytometry. Representative examples of healthy and ruptured tendon derived cultures are shown. The percentage of cells in the G0/G1, S and G2/M phases are similar in the two groups composed by tenocytes derived from healthy or ruptured tendons (Student T test: ∧p = not significant). Results reported in graph represent the mean values ±SE obtained from three independent experiments.

Explants of human tendons derived from 4 healthy (semitendinosus) and 4 ruptured (Achilles) sources: all patients affected by ruptures and selected for the explant have been accompanied by a previous story of Achilles chronic degenerative tendinopathy. Cells were then plated and grown approximately 10 days in culture flasks until started to form aggregates and constitute a monolayer. As we previously reported (Vetrano et al., 2011) [24], they exhibited several patterns of cell morphology, which probably depend on the various levels of cell differentiation and do not appear to be related to the different source of the explants. All experiments were carried out at the passage P1. To molecularly characterize the cell cultures, RT-PCR was used to measure the gene expression of typical tenocyte markers [14], [15], [18], [19], i.e. type I and III Collagens (Col I-III), Scleraxis (Scx), Tenomodulin (Tnm) and Tenascin-C (Tn-C). The molecular analysis permitted to detect in our samples the simultaneous expression of all gene markers, assessing the quality and purity of our cell cultures, independently by the different source of the explants. (Fig. 1A).

Comparing the quantitative expression among the two groups of cultures (healthy and ruptured), we found significantly different levels of Col I and Scx, which were clearly higher in cells from pathological compared to healthy samples (p<0.05) (Fig. 1A). In contrast, although variable among the cell cultures, the mean transcript levels of Tnm and Tn-C were not significantly different (Fig. 1A). Surprisingly, the levels of Col III were comparable in healthy and pathological derived cultures, which is not in agreement with some published studies [7], [27].

To verify possible differences in the proliferative behaviour of the healthy and ruptured tendon-derived cultures, we evaluated the growth rate characteristics analyzing the cell cycle distribution by cytofluorimetry, as described in Materials and Methods. The analysis of the cell cycle phase distribution, performed by DNA staining using fluorescent PI, showed that the two groups of cultures were characterized by a similar percentage of cells in the G0/G1, S and G2/M phases (Fig. 1B). Representative examples for cell cycle of tendon derived cultures are shown in Fig. 1B. The statistical analysis revealed no significant differences in mitotic ability of cell cultures (mean values of G2/M phase: 10,2% if derived from healthy vs 11,6% if derived from ruptured tendons, p = not significant; Fig. 1B).

### ESWT-mediated Effects of the Cultured Tenocytes

**Figure 2:**
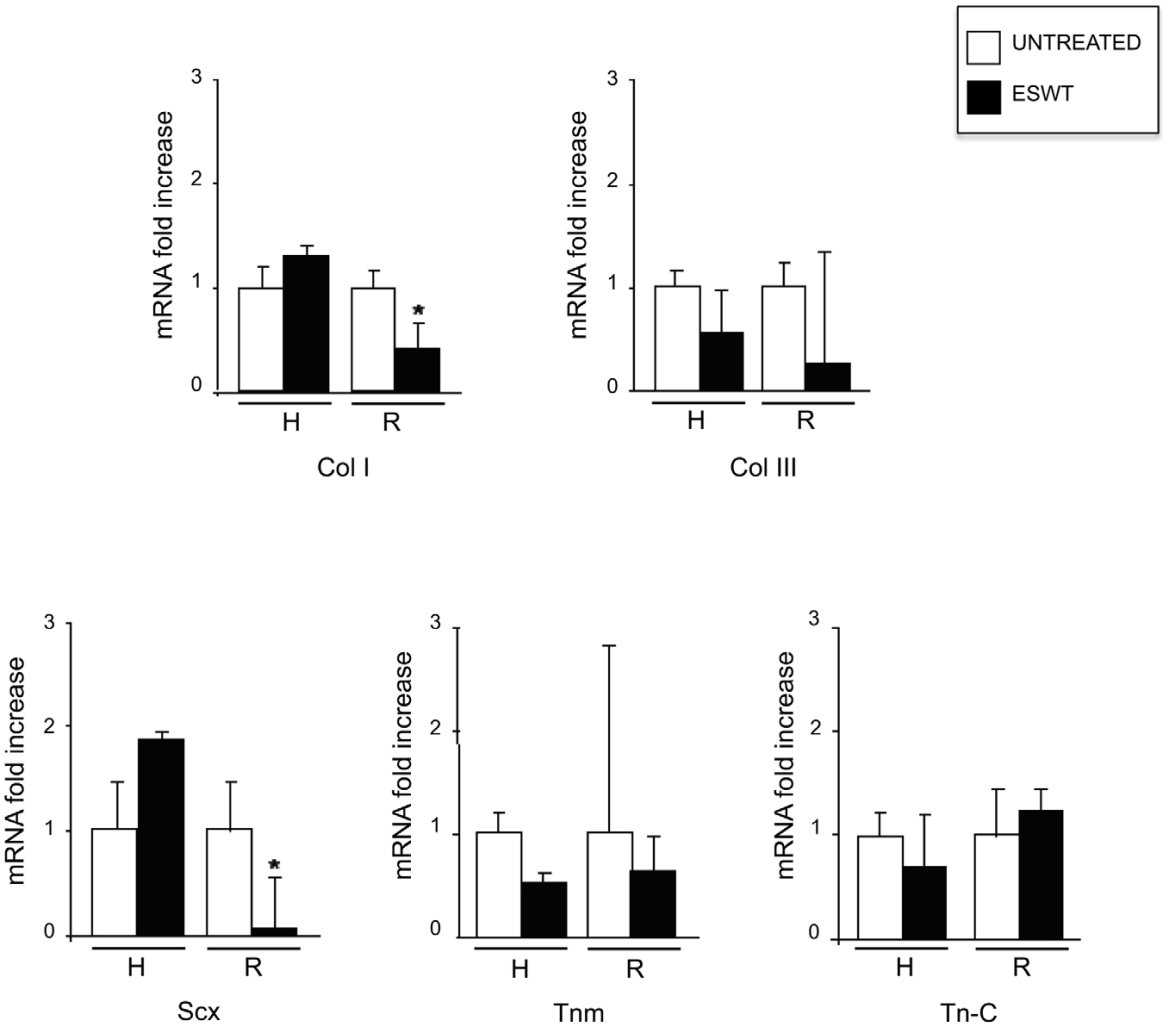
Expression of tenocyte markers in cultures derived from healthy and ruptured tendons in response to ESWT. Tenocytes derived from healthy (H) and ruptured (R) tendons were exposed to ESWT, incubated for 24 hours at 37°C before mRNA extraction. Unexposed cells were used as control. The molecular analysis reveals that the ESW treatment induces a significant decrease in the expression of Col I and Scx in cells from ruptured respect to healthy samples. No significant differences are observed for the other gene markers (Col III, Tnm and Tn-C) after ESWT in both groups of H and R cultures. Results reported in graph represent the mean values ±SE obtained from three independent experiments. Student T test was performed and significance level has been defined as described in materials and methods: *p>0.05 vs the corresponding untreated.

To analyze the effects of ESWT on the two groups of tenocytes obtained from healthy or ruptured sources, we treated the cultures at the dose of 0.14 mJ/mm^2^ (1000 impulses), previously selected for the clinical efficacy in vivo, with no interference in the *in vitro* cell viability [24]. RT-PCR analysis on the cultures exposed to ESWT was performed as above, normalizing each sample to the correspondent untreated control. As shown in Fig. 2, comparing the patterns of mRNA expression in healthy and ruptured tendon-derived cells, the molecular analysis showed that the treatment induced a significant decrease in the expression of Col I and Scx (p<0.05) only, whereas no significant differences have been detected for the other markers (Fig. 2).

Taking together, the molecular results demonstrate that the most evident effects of ESWT are related to a reduction of the expression of the same two markers (Col I and Scx) which were higher -before treatment- in the ruptured versus healthy cultures (Fig. 1A). These data appear in accordance with previous reports showing an early decrease in the expression of differentiative markers in response to other treatments for tendinopathies [28], [29].

### ESWT-mediated Functional Activities of the Tenocyte Cultures

We and others have reported that ESWT is able to enhance *in vitro* cellular activities, such as proliferation, which could probably contribute to tendon repair *in vivo* and seem to be imputable to shock wave-induced variations in the morphological appearance of the cells [24], [30], [31]. Therefore, we sought to analyze if the exposure to ESWT would exert similar or different effects on the morphology as well as on the growth baseline activity in healthy compared to ruptured sources. However no ESWT-induced morphological variations in tenocytes derived from healthy compared to ruptured tendons have been detected (data not shown).

**Figure 3:**
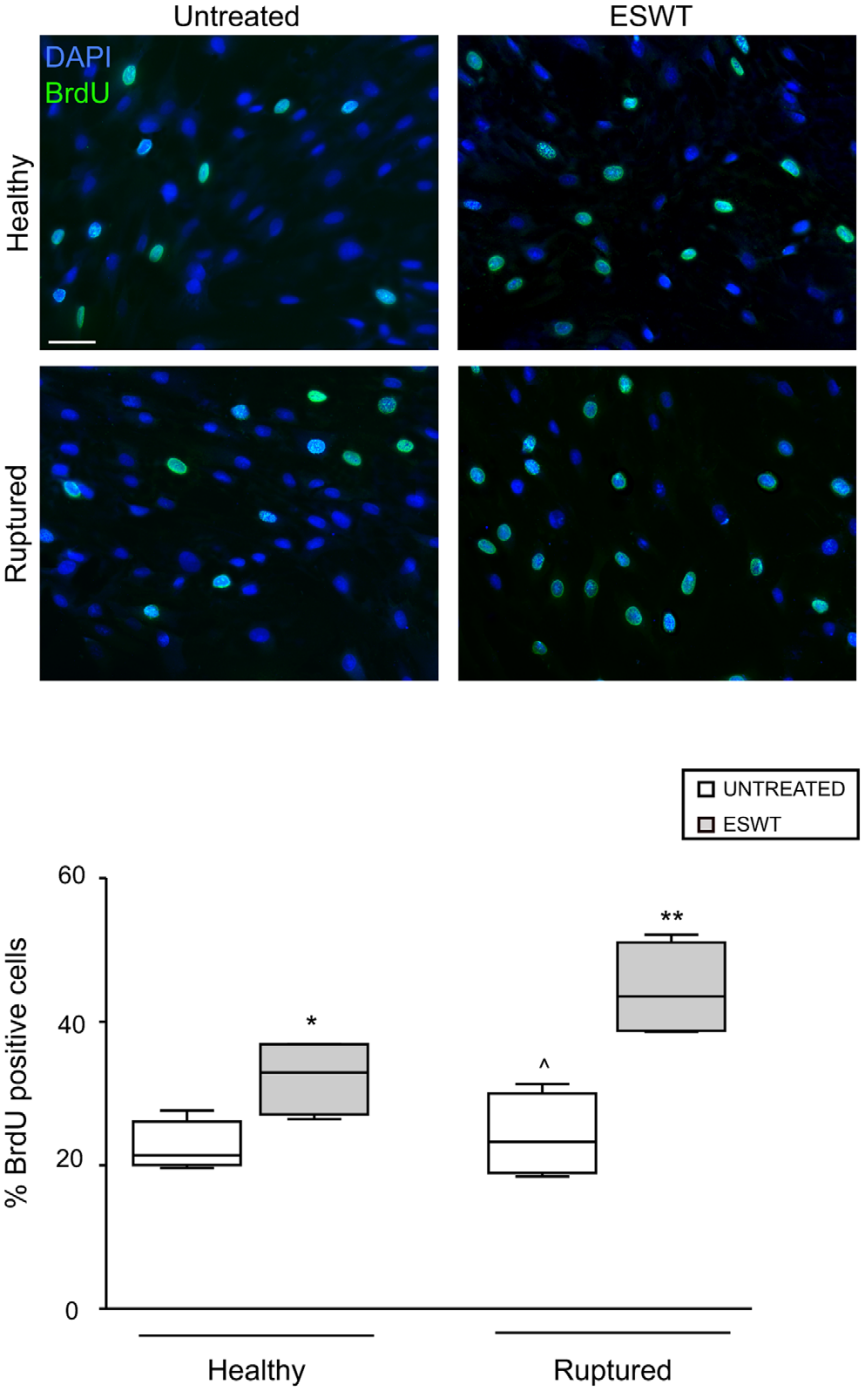
BrdU incorporation assay of tenocytes cultures in response to ESWT. Tenocytes derived from healthy and ruptured tendons were exposed to ESWT, incubated for 96 hours a 37°C, treated with BrdU during the last 4 hours of incubation, fixed and stained with anti-BrdU antibodies. Unexposed cells were used as control. Quantitative analysis was performed by counting, for each cultures, the percentage of BrdU-positive nuclei (green) on a total of at least 300 cells, randomly observed in 10 microscopic fields from three different experiments. Nuclei are stained with DAPI. Bars: 20 µm. The box plots show the average percentages of BrdU positivity obtained from three independent experiments for each culture. The median value of the group is indicated by the middle horizontal line. Mann-Whitney test: *p>0.05 vs the corresponding untreated; **p>0.05 vs the corresponding untreated and p>0.05 vs treated healthy; ∧p = not significant vs untreated healthy.

In addition, to evaluate the proliferative rate of our tenocyte cultures in response to ESWT, we performed a BrdU assay, focusing on the possible differences between cells derived from healthy compared to pathological tendons. All cells were incubated with BrdU, then fixed and stained with anti-BrdU antibodies, to identify DNA synthesizing proliferating cells, whose nuclei were stained with DAPI (Fig. 3). Quantitative analysis of the percentage of cells presenting BrdU-positive nuclei indicated that ESWT was able to significantly increase the proliferation of either healthy or ruptured tendon-derived cells, when compared to untreated controls (p<0.05). Interestingly, the shock wave mediated effects were more evident in tenocytes explanted from ruptured versus healthy tendons, since the ratio of the proliferative activity was 1.75 fold increased (Fig. 3).

**Figure 4:**
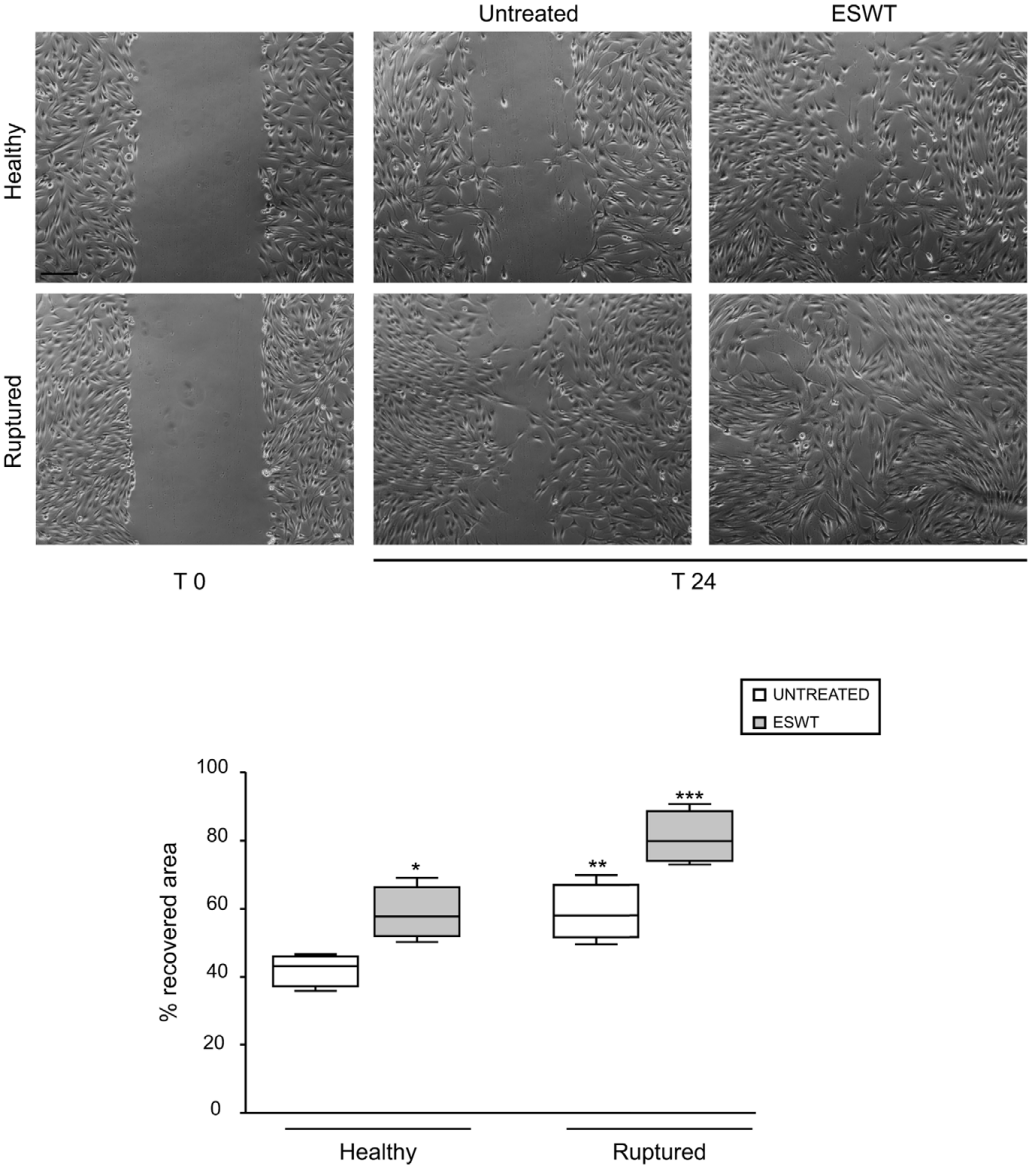
Scratch assay on tenocytes cultures in response to ESWT. Tenocytes derived from healthy and ruptured tendons were exposed to ESWT and incubated for 48 hours a 37°C. A cell-free scratched area was introduced in confluent cultures as described in materials and methods. The cells were allowed to migrate for 24 hours and then photographed under phase contrast microscopy. Unexposed cells were used as control. The images are representative of three independent experiments. In ESW treated cultures, many cells in the scratched area display a typical migratory phenotype. Cell migration was evaluated by measuring the repopulation of the cleared area: the box plots show the average percentages of recovered area obtained from three independent experiments for each culture. Bars: 50 µm. The median value of the group is indicated by the middle horizontal line. Mann-Whitney test: *p>0.05 vs corresponding untreated; **p>0.05 vs untreated healthy; ***p>0.05 vs corresponding untreated and p>0.05 vs treated healthy.

In order to further evaluate the repairing capacity of the exposed cultured tenocytes, we next performed a classical scratch test of cell migration, able to mimic a possible ESWT-induced *in vitro* wound repair. The cell migration was quantified measuring the mean of recovered areas after scratching, as described in Material and Methods. The shock wave exposure induced a typical migratory phenotype in the majority of the tenocytes and this effect was especially evident for cells derived from ruptured tendons. As shown in Fig. 4, the untreated cells -when obtained from ruptured compared to healthy tendon- showed a significant increase of motility. Quantitative analysis clearly indicated that the ESWT-triggered cell motility was significantly higher compared to untreated control (p<0.05) and was more evident in cells from ruptured versus healthy tendons (ratio of migratory activity = 1.3; p<0.05) (Fig. 4).

## Discussion

We previously reported that human tenocytes derived from healthy semitendinosus tendon displayed heterogeneous patterns of differentiated phenotypes and that ESWT induced a significant increase in cell proliferation and Col I synthesis [24]. In the present study, we aimed to extend this model comparing tenocytes derived from healthy to ruptured tendons, in order to establish whether the functional activities (i.e. proliferation, migration and differentiation) of such cells could be attributed to the different source of the explants and influenced by the ESWT.

Tenocyte characterization was performed by the molecular analysis of typical tenocyte markers -which were simultaneously expressed in all our cultures- and by the evaluation of the cell cycle phase distribution. Although the growth rate analysis displayed no significant differences in the two groups of cultures, at molecular level, tenocytes derived from healthy tendons showed baseline levels of Col I and Scx lower compared to cultured cells explanted from ruptured tendons, whereas no substantial variations related to the different sources of the explants in Col III, Tnm and Tn-C have been found. We might suppose that in ruptured tendon a repairing program -which requires an increase in collagen production- has been naturally triggered by the injury. Concerning Col I-III expression, our results are apparently not in agreement to previous report [7], where a decrease in Col I and an increase in Col III delivery in cultured tenocytes obtained from ruptured compared to normal tendon was observed. However, the comparison between this report and our study is difficult, because here we evaluated gene expression, whereas the previous report analyzed the Col I-III production by an immunostaining approach to visualize collagen positive cells [7]. In addition, prior molecular observations performed on ruptured tendons showed controversial results [17], [32], which could be explained by the different passages, days of culture and explant origin, characterizing the human tenocytes *in vitro*. Taken together, our data could be ascribed to a possible activation -in the cells from injured tendon- of an early differentiation program, as demonstrated by the increase in the expression of the early marker Scx, whereas the later differentiation marker Tnm was not yet modified. Nevertheless, we assume that Tnm and Col III may probably increase their expression in a following phase of the differentiation program (as previously shown by Docheva et al., 2005; Shukunami et al., 2006; Luo et al., 2009) [14], [15], [33].

When we applied ESWT to our cultured cells, the levels of Col I and Scx were significantly diminished in ruptured compared to healthy tendon-derived cells, indicating that tenocytes obtained by injured explants (differently from healthy) are impaired by the treatment to undertake their differentiation program. This is in agreement with previous observations [12], [28] suggesting that this ESWT-mediated delay of cell differentiation may be a necessary effect required for tendon regeneration, because treated tenocytes are probably more inclined to undergo proliferation and migration with limited differentiation. In accordance with this hypothesis on the shock wave-mediated functional activities of human cultured tenocytes, we found -for the first time at our knowledge- that ESWT was significantly able to increase the proliferative and migratory activities of either healthy or ruptured tendon-derived compared to untreated human cells. Nevertheless, the shock wave efficacy was more evident in tenocytes explanted from ruptured than from healthy tendons: in particular, BrdU assay ascertained that the ratio of proliferative activity was 1.75 folds increased in ruptured then healthy tendon-derived cultured tenocytes, whereas scratch test of cell migration depicted an increment of 1.3 folds showing a typical migratory phenotype in the treated samples. We suggest that those different biological behaviours may depend on the distinct sources of the explants and could be probably related to the *in vitro* wound healing capacity of the ESWT-induced human cultured tenocytes. This shock wave-mediated increase in repairing capacity of the tenocytes explanted from ruptured tendons could explain the encouraging benefits of shock wave therapy -applied at the dose of 0.14 mJ/mm^2^ energy level and 1000 impulses- on tendinopathic patients [34].

We are conscious of the limitations of the present study, since we compared cultured tenocytes derived from different sources of tendons with a limited number of samples. However, we think that our experimental model could be considered reasonable, because our cell characterization, performed by morphological, molecular and functional analysis, showed no significant differences between Achilles and semitendinosus tendon-derived cultures, similarly to the results previously reported by others [35].

In addition, regarding the possibility of using cells explants of healthy Achilles tendon specimens obtained from cadaver material [36], even not considering the difficulties to retrieve enough material to establish a satisfying number of tissue-derived cell cultures, we pondered that cultures obtained by cadaver material can be also considered uncomparable to explants by living patients. Moreover, concerning the possible use of amputated legs, as in the case of established cultures from material derived from patients (not healthy) who underwent amputation for peripheral vascular disease [7], we are not sure that this model may be useful for our purpose since one of the main goal of our project was to evaluate the potential of ESWT to increase tendon healing, whose effectiveness may be influenced by a correct vascularization [37], [38].

At the light of our observations, performed on healthy as well as on ruptured tendons, and considering the complexity of the healing process -including well known recognized factors involved such as growth factors and metalloproteinases [39]–[42]- further investigations are required to evaluate the effects of ESWT on the biological activities of tenocytes derived from tendinopathic tendons (according to Maffulli et al., 2000) [7], which probably represent the *in vivo* natural target of this therapy.
